# What can we learn from real world studies on direct oral anticoagulants?

**DOI:** 10.1007/s12471-017-1029-5

**Published:** 2017-08-03

**Authors:** M. Coppens

**Affiliations:** Department of Vascular Medicine, Academic Medical Center, Amsterdam, The Netherlands

Direct oral anticoagulants (DOACs) are replacing vitamin K antagonists (VKA) for most patients with atrial fibrillation (AF) and venous thromboembolism. The four currently available DOACs were compared with VKA in phase III randomised controlled trials that collectively included over 100,000 patients. The results show that DOACs, at a fixed dose without laboratory monitoring, are at least as effective as VKA therapy and reduce the risk of major bleeding, including a 50% risk reduction of intracranial bleeding [1]. As a consequence, DOACs are now recommended over VKA in the international guidelines [2]. Yet, after regulatory approval there were concerns that the trial results may not apply to the patients in our daily clinical practice that generally have more comorbidities than a standard trial population. This led to a broad call for post-authorisation (phase IV) studies that were soon referred to as Real World studies, suggesting that the phase III studies originate from a world that is unreal.

Indeed, it was previously shown that bleeding risk on oral anticoagulation is higher for patients who would have been excluded from randomised trials compared with patients who meet all the eligibility criteria [3]. However, the eligibility criteria of the phase III trials were rather liberal allowing inclusion of patients with significant comorbidities. For example, the proportion of elderly patients (75 years or over) was 38%, half of patients had a CHADS_2_ score of 3 or higher, 30% of patients had suffered a prior stroke or TIA, and 19% of patients had chronic kidney damage with an eGFR of 30–50 ml/min [1]. At first glance this already greatly resembles most of our clinical practices. The appendices of the trials further show that over 80% of patients screened for participation were actually enrolled into the trials, again suggesting that the enrolled population is representative of daily practice.

We are currently facing a tsunami of so called Real World studies. As practising clinicians, it is tough to keep up and to appreciate the lessons that can be learned and, equally important, what should be interpreted very cautiously. While all studies are referred to as Real World studies, they include very different types of studies. First are prospective, non-interventional cohort studies, in which patients are actively enrolled, specific eligibility criteria are applied, comorbidities are extensively collected and outcomes verified by independent adjudication committees. Next are data from registries that are less controlling regarding eligibility criteria and aim to include all consecutive patients. Outcomes are usually not centrally adjudicated and thereby slightly less reliable. Finally, the bulk of Real World studies originate from insurance claim databases using disease specific billing codes linked with drug prescription records. The advantage of such studies is the large number of patients and routine follow-up, arguably creating the best reflection of patients from our daily clinical practice. However, quality of data is an important concern. The accuracy of coding is variable and is not easily verified, patient characteristics such as renal function and body weight are often not collected and finally, only outcomes that lead to hospital visits and new billing codes can be captured.

All thus far published non-interventional studies share one feature: patients treated with DOACs are younger and have less comorbidities than patients on VKA therapy. This may reflect our reluctance as doctors to put our highest risk patients on a new class of drugs first. However, this introduces significant bias (confounding-by-indication) when comparing different drugs. The differences between treatment groups can be partly accounted for by propensity score matching. With this statistical technique treatment effects are estimated by accounting for the patient characteristics that predict receiving a specific treatment. The validity of this technique is critically dependent on the availability and reliability of the collected variables. Missing or unknown variables that were simply not collected (most often precise renal function) will lead to residual confounding that cannot be accounted for. Therefore, comparisons in non-interventional studies, even with careful propensity score matching, remain less reliable than comparisons from randomised controlled trials. In fact, we need to be reminded that the true purpose of post-authorisation studies is to evaluate prescription patterns, to monitor patient adherence and quality of life and to evaluate rare long-term outcomes for which the follow-up of the randomised trials was simply too short. Additionally, post-authorisation studies can evaluate the effects of interventions in previously unstudied populations. Patients with stage IV renal failure (eGFR 15–30 ml/min) are of particular interest as these patients were excluded from the randomised studies, but regulatory approval was nevertheless obtained for direct factor Xa inhibitors based on population pharmacokinetic modelling.

In this edition of the Netherlands Heart Journal, Pisters and colleagues report on the subgroup of Dutch patients who were enrolled in the XANTUS study [4]. The authors have to be commended for focusing on the points that should be taken from such studies. Dutch AF patients seem to have lower CHA_2_DS_2_-VASc scores compared with patients from other countries, likely resulting from a larger proportion of patients with paroxysmal AF receiving therapy. Importantly, label-discordant dosing was observed in 8% of the total cohort and 33% of patients treated with 15 mg rivaroxaban had a normal renal function. In 36% of patients renal function was unknown, which is surprising considering dosing of rivaroxaban is determined by renal function. Finally, patient adherence seemed good with 14% permanent discontinuation and the incidence rates of bleeding and mortality seem to be consistent with the previous randomised studies.

The future for patients on oral anticoagulation has improved significantly over the last years. The rapidly emerging Real World studies are answering additional questions and seem to be confirming the phase III results solidifying the body of evidence of DOACs. However, we should stop referring to them as Real World studies, as this term wrongfully devaluates the results of robust randomised phase III studies that have shown very consistent results in key subgroups such as the elderly and patients with impaired renal function. We should consider referring to these studies by what they are: Practice-Based Studies, providing useful and needed additional information, but inherently inferior to randomised controlled trials when it comes to drug comparisons.
